# Epigenetic and post-transcriptional repression support metabolic suppression in chronically hypoxic goldfish

**DOI:** 10.1038/s41598-022-09374-8

**Published:** 2022-04-02

**Authors:** Elie Farhat, Giancarlo G. M. Talarico, Mélissa Grégoire, Jean-Michel Weber, Jan A. Mennigen

**Affiliations:** Department of Biology, University of Ottawa, 10 Marie Curie, Ottawa, ON K1N 6N5 Canada

**Keywords:** Epigenetics, Non-coding RNAs, Transcription, Metabolism

## Abstract

Goldfish enter a hypometabolic state to survive chronic hypoxia. We recently described tissue-specific contributions of membrane lipid composition remodeling and mitochondrial function to metabolic suppression across different goldfish tissues. However, the molecular and especially epigenetic foundations of hypoxia tolerance in goldfish under metabolic suppression are not well understood. Here we show that components of the molecular oxygen-sensing machinery are robustly activated across tissues irrespective of hypoxia duration. Induction of gene expression of enzymes involved in DNA methylation turnover and microRNA biogenesis suggest a role for epigenetic transcriptional and post-transcriptional suppression of gene expression in the hypoxia-acclimated brain. Conversely, mechanistic target of rapamycin-dependent translational machinery activity is not reduced in liver and white muscle, suggesting this pathway does not contribute to lowering cellular energy expenditure. Finally, molecular evidence supports previously reported chronic hypoxia-dependent changes in membrane cholesterol, lipid metabolism and mitochondrial function via changes in transcripts involved in cholesterol biosynthesis, β-oxidation, and mitochondrial fusion in multiple tissues. Overall, this study shows that chronic hypoxia robustly induces expression of oxygen-sensing machinery across tissues, induces repressive transcriptional and post-transcriptional epigenetic marks especially in the chronic hypoxia-acclimated brain and supports a role for membrane remodeling and mitochondrial function and dynamics in promoting metabolic suppression.

## Introduction

Environmental hypoxia is a widely occurring environmental phenomenon that can be lethal to most animals. Severe environmental states of O_2_ limitation (severe hypoxia or anoxia), are comparatively rare and the capacity for metabolic suppression to survive these conditions evolved in few animals^1^. It can be achieved through transcriptional, post-transcriptional, translational, and post-translational modifications as well as epigenetics^2,3^. These mechanisms allow for the downregulation of energetically costly processes such as (i) Na^+^/K^+^-ATPase, (ii) the oxygen-sensing machinery^4^, (iii) transcription and (iv) protein synthesis^5^ by repressing processes such as the mechanistic target of rapamycin (m-TOR) signaling pathway^6^. Here, we test the hypothesis that molecular cellular hypoxia sensing pathways are activated in several tissues in goldfish exposed to chronic hypoxia and that transcriptional and post-transcriptional epigenetic mechanisms contribute to the inhibition of energetically costly cellular processes underlying metabolic suppression.

The oxygen-sensing machinery functions via enzymes, such as the egl nine homologue gene family (*Egln*) encoded prolyl hydroxylase domain-containing proteins (PHD). Of these, both *Egln1* and *Egln3* (coding for PHD2 and 3), but not *Egln2* (coding for PHD1), immediately respond to intracellular decreases in O_2_^7–9^. Additionally, noncoding transcripts such as microRNA (miRNA) have emerged as transcriptional markers and mediators of physiological responses to hypoxia^10–13^. As such, *miRNA-210-5p* has been characterized as the master hypoxia-responsive miRNA (hypoxamiR)^13,14^. Transcription and translation are molecular level energy sinks that are regulated by hypoxia^15,16^. Indeed, consequences of hypoxia exposure on global DNA methylation levels and gene expression of enzymes involved in DNA methylation dynamics/turnover [the de novo writer, DNA methyltransferase 3 (*Dnmt3*) and eraser ten-eleven translocation methylcytosine dioxygenases (*Tet*)] have been well described in cancer research^17^. Moreover, hypoxia regulates components of the canonical miRNA biogenesis machinery^18,19^, supporting the notion that hypoxia may globally affect (post-) transcriptional regulation of gene expression via epigenetic regulation.

Goldfish are champions of hypoxia tolerance that suppress their metabolic rate by up to 74% to cope with chronically low O_2_^20–22^. This hypometabolic state occurs together with tissue-specific downregulation of Na^+^/K^+^-ATPase, a decrease in mitochondrial density but an increased reliance on brain lipid oxidation during chronic hypoxia^23^. Moreover, goldfish exposed to severe hypoxia for 12 h exhibit metabolic suppression to stimulate AMP-activated protein kinase (Ampk) to support the necessary downregulation of liver protein synthesis^24^. We have also previously shown that changes in membrane lipid composition, particularly cholesterol abundance, may contribute to hypometabolism in goldfish^20^ as in naked mole-rats^25^ during chronic hypoxia. However, molecular, and especially epigenetic, foundations underlying such hypoxia tolerance in goldfish tissues under metabolic suppression are currently not well understood. Therefore, we measured the responses of various goldfish tissues (brain, liver, white muscle, heart) at normoxia (21 kPa PO_2_) and, following gradual reduction to severe hypoxia (2.1 kPa PO_2_) over the course of a week, maintained chronic hypoxia from 1 to 4 weeks on the following parameters: (i) Relative transcript abundance of *egln1, egln3* and *miRNA-210-5p* transcripts orchestrating the oxygen-sensing machinery; (ii) relative abundance of transcripts encoding epigenetic pathway machinery and global epigenetic markers (DNA methylation) that could suggest transcriptional (*dnmt3*; *tet1-3*) and post-transcriptional [DiGeorge syndrome critical region 8 (*dgcr8*), *dicer*, exportin 5 (*xpo5)*, argonaut 2 (*ago2*)] suppression of energetically costly gene expression at the genome level; (iii) post-translational activation status of the energy sensor AMP-kinase subunit alpha (Ampkα) and mechanistic target of rapamycin (mTor)-dependent energetically costly protein synthesis pathway components [ribosomal protein S6 (S6), phosphorylated eukaryotic translation initiation factor 4E-binding protein 1 (4e-bp1) and phosphorylated protein kinase B (Akt)] and (iv) relative abundance of transcripts with roles in cholesterol metabolism [3-hydroxy-3-methylglutaryl-Coenzyme A synthase 1 (*hmgcs1) and miRNA-122-5p* involved in cholesterol synthesis, the cholesterol sensor liver x receptor (*lxr*) and the cholesterol degradation pathway component cholesterol 7alpha-hydroxylase (*cyp7a*)], the mitochondrial lipid oxidation enzyme carnitine palmitoyltransferase 1A (*cpt1a*) and mitochondrial fusion [mitofusins 1 and 2 (*mfn1*, *mfn2*)] and fission [mitochondrial fission protein 1 (*fis1*)] proteins, as membrane cholesterol and mitochondrial plasticity have been identified to contribute to metabolic suppression in chronically hypoxic goldfish in two recent companion studies^20,23^.

## Results

### Chronic hypoxia robustly induces components of oxygen-sensing machinery in goldfish

**Figure 1:**
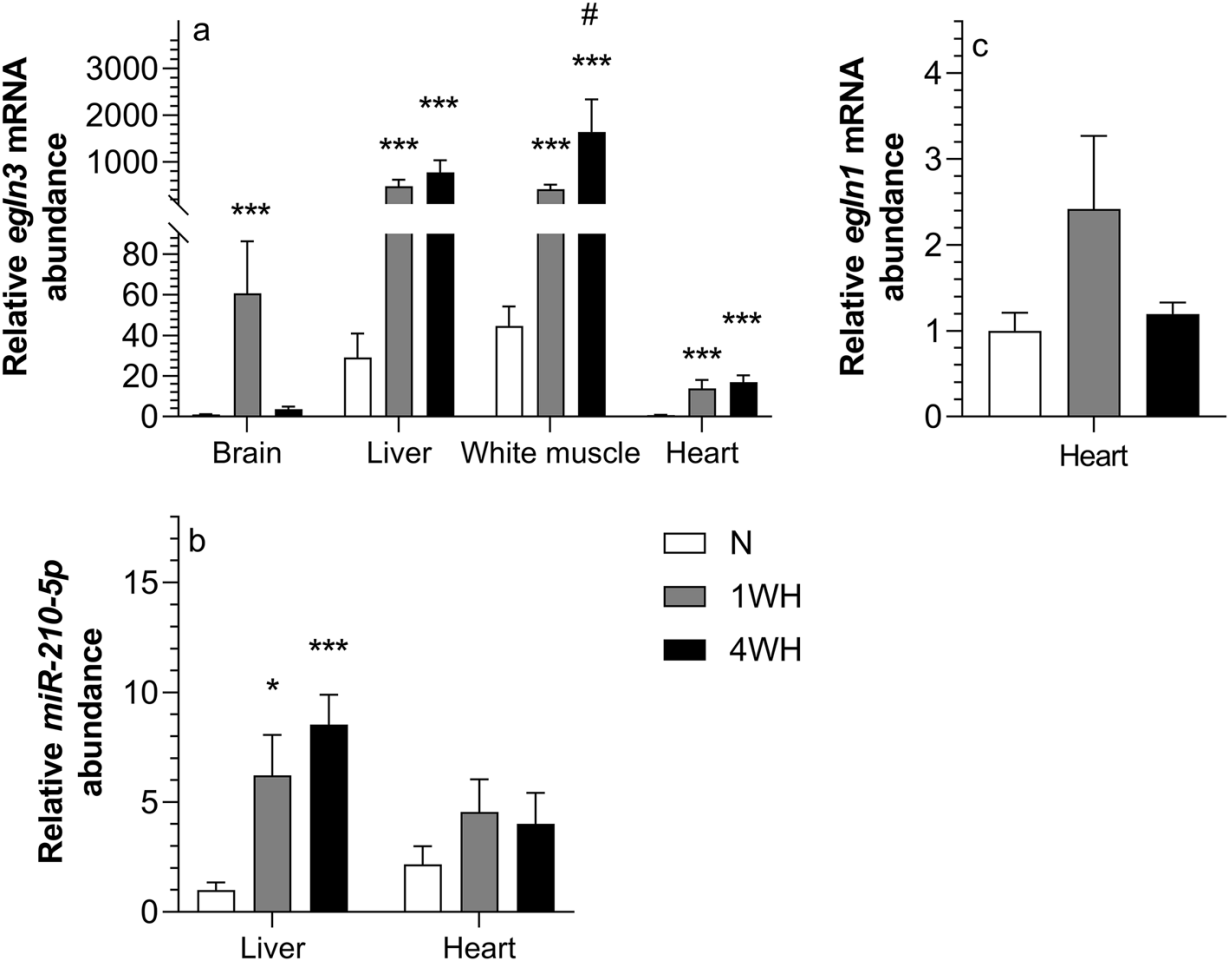
Relative abundance of mRNA targets involved in the oxygen-sensing machinery in tissues of normoxic controls (N), 1-week hypoxic (1WH) and 4-week hypoxic (4WH) goldfish presented in panel (**a**) *(egln3*), (**b**) (*miRNA-210-5p*) and (**c**) (*egln1*). Values are means ± standard error of the mean (s.e.m.); sample size = 7–12 per group. Differences between normoxia and chronic hypoxia are indicated as *(*p* < 0.05) and ***(*p* < 0.001). Differences between 1 and 4WH are indicated as #(*p* < 0.05).

The relative transcript abundance of *egln3* was higher in 1WH and 4WH of all tissues (vs normoxia; *p* < 0.001) except brain where it was only higher in 1WH (*p* < 0.001). In comparison to 1WH, the relative transcript abundance of *egln3* was higher in 4WH white muscle (WM) (*p* = 0.041) without changing in the other tissues (*p* > 0.05) (Fig. 1a). Moreover, relative transcript abundance of *miRNA-210-5p* was higher in both 1WH (*p* = 0.015) and 4WH (*p* < 0.001) liver (both vs normoxia) but remained unchanged in heart (*p* > 0.05) (Fig. 1b). Finally, relative *egln1* transcript abundance was unchanged in 1WH and 4WH heart (*p* > 0.05) (Fig. 1c).

### Chronic hypoxia time-dependently regulates DNA methylation in highly oxidative tissues

**Figure 2:**
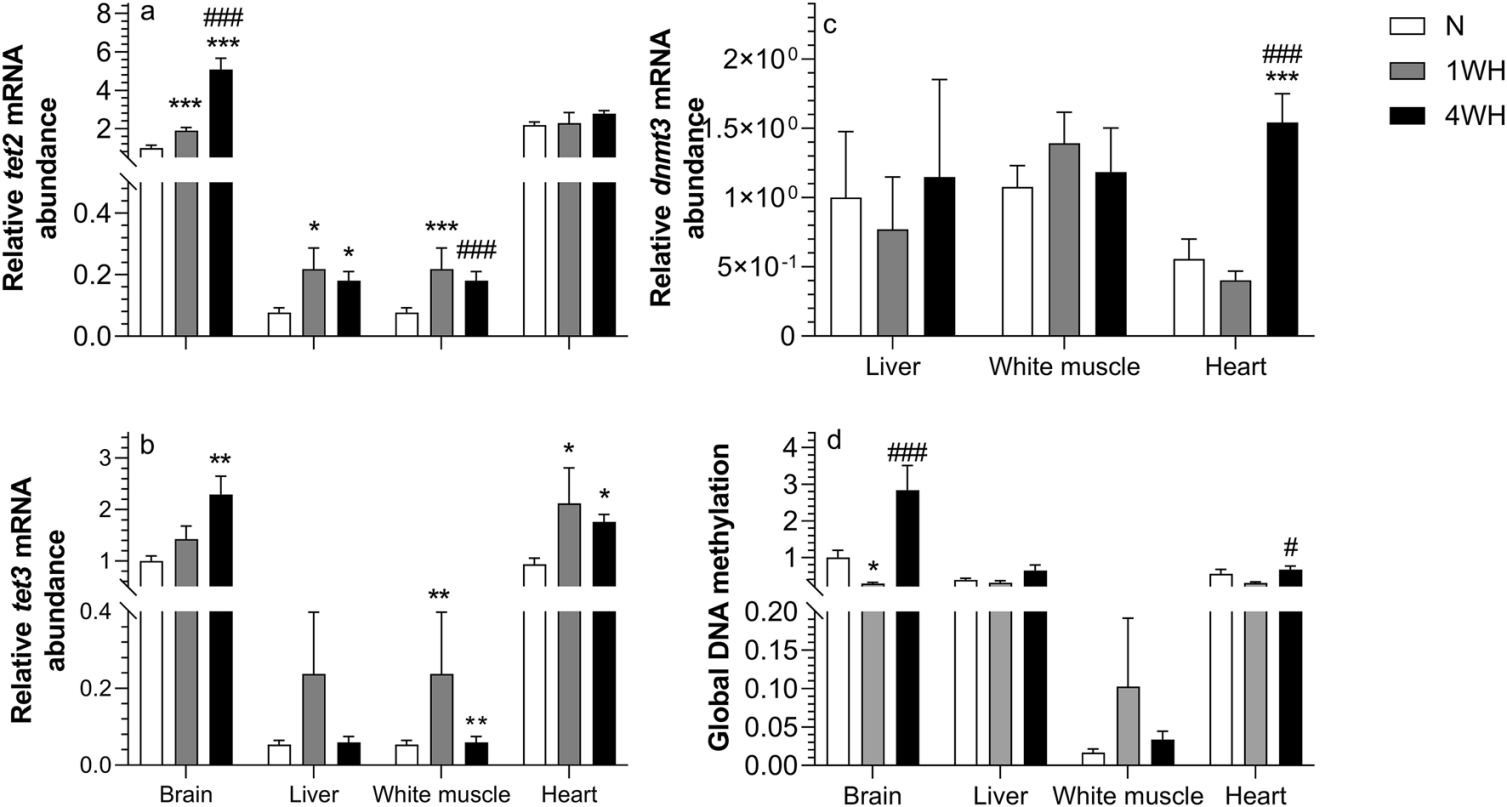
Relative abundance of transcripts involved in DNA methylation dynamics are presented in panels (**a**) (*tet2*), (**b**) (*tet3*) and (**c**) (*dnmt3*), and global DNA methylation is presented in panel (**d**). In all panels, measures in tissues of normoxic controls (N), 1-week hypoxic (1WH) and 4-week hypoxic (4WH) goldfish are presented. Values are means ± standard error of the mean (s.e.m.); sample size = 7–12 per group. Differences between normoxia and chronic hypoxia are indicated as *(*p* < 0.05), **(*p* < 0.01) and ***(*p* < 0.001). Differences between 1 and 4WH are indicated as #(*p* < 0.05) and ###(*p* < 0.001).

Relative transcript abundance of *tet2* was higher in 4WH brain (vs normoxia and 1WH; *p* < 0.001) and liver (vs normoxia; *p* = 0.016), but lower in white muscle (vs 1WH; *p* < 0.001) without changing in heart (*p* > 0.05). Moreover, relative *tet2* transcript abundance in 1WH was increased in brain (*p* < 0.001), liver (*p* = 0.024) and white muscle (*p* < 0.001) when compared to the normoxic controls, without changing in heart (*p* > 0.05) (Fig. 2a). Relative transcript abundance of *tet3* was higher in 4WH brain (vs normoxia; *p* = 0.008), white muscle (*p* = 0.011) and heart (*p* = 0.009) (both vs normoxia). Relative transcript abundance of *tet3* was also higher in 1WH white muscle (*p* = 0.007) and heart (*p* = 0.019) when compared to normoxia. There was no effect of either 1WH or 4WH on relative *tet3* transcript abundance in the liver (*p* > 0.05) (Fig. 2b). Finally, relative transcript abundance of *dnmt3* was only increased in 4WH heart (vs normoxia and 1WH; *p* < 0.001), without changing in the other tissues (*p* > 0.05) (Fig. 2c). Global DNA methylation was increased in 4WH brain (vs 1WH; *p* < 0.001) and heart (vs 1WH; *p* = 0.047) without changing in this group in other tissues (*p* > 0.05). However, methylation was decreased in 1WH brain (vs normoxia; *p* = 0.02), without changing in the other tissues (*p* > 0.05). There were no effects of chronic hypoxia (1WH or 4WH) on DNA methylation in either liver or white muscle (*p* > 0.05) (Fig. 2d).

### miRNA biogenesis pathway is induced in chronic hypoxia acclimated brain

**Figure 3:**
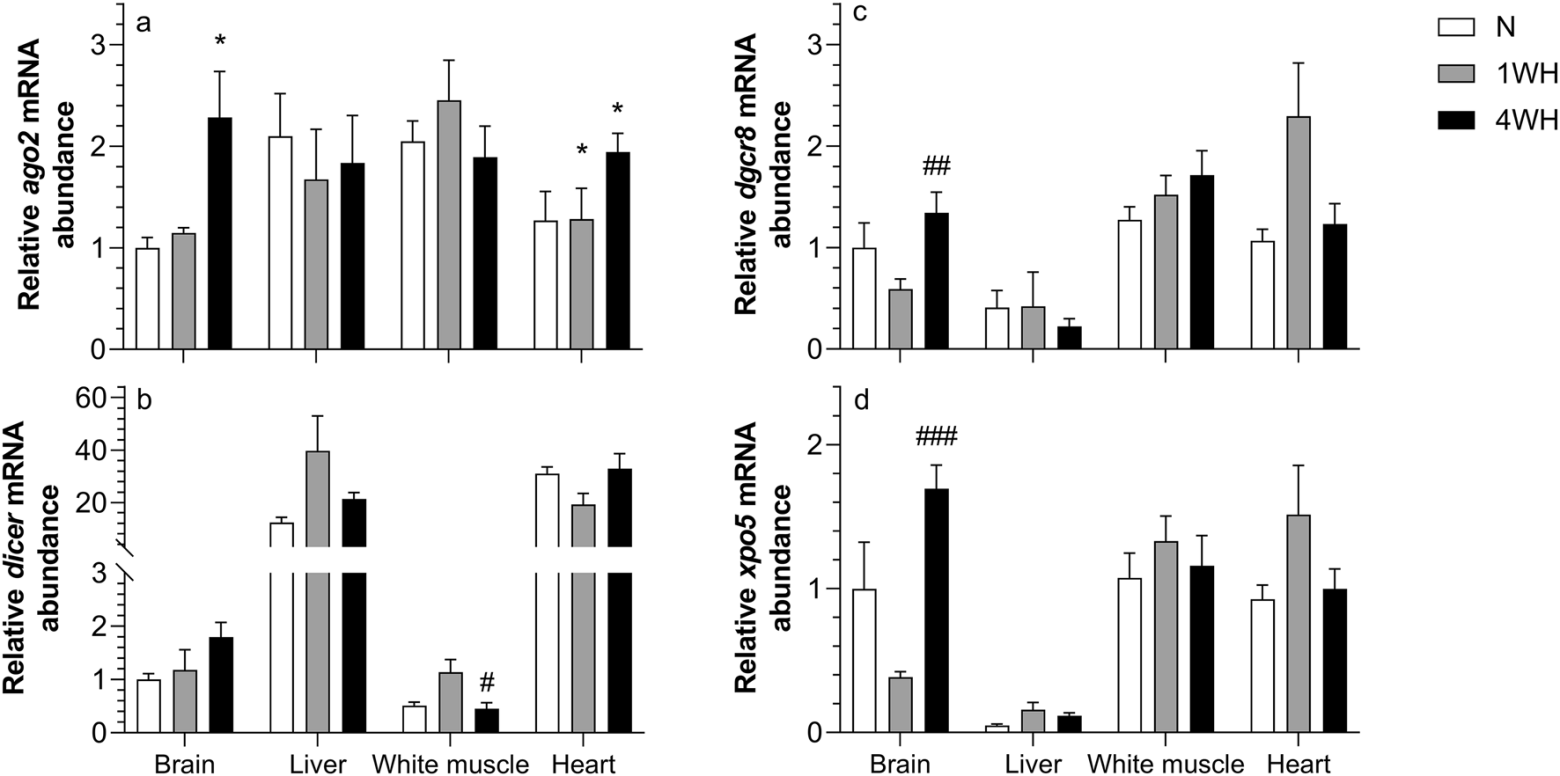
Relative abundance of transcripts involved in the canonical miRNA biogenesis pathway (*ago2*, panel (**a**); *dicer*, panel (**b**); *dgcr8*, panel (**c**); *xpo5*, panel (**d**)) in tissues of normoxic controls (N), 1-week hypoxic (1WH) and 4-week hypoxic (4WH) goldfish. Values are means ± standard error of the mean (s.e.m.); sample size = 7–12 per group. Differences between normoxia and chronic hypoxia are indicated as *(*p* < 0.05). Differences between 1 and 4WH are indicated as #(p < 0.05), #(p < 0.01) and ###(p < 0.001).

Relative transcript abundance of *ago2* was higher in 4WH brain (vs normoxia; *p* = 0.025) and heart (vs normoxia; *p* = 0.042 and 1WH; *p* = 0.046), but it remained constant in the other tissues (*p* > 0.05) (Fig. 3a). Relative transcript abundance of *dicer* increased in 4WH white muscle (vs 1WH; *p* = 0.02), without changing in other tissues (*p* > 0.05) (Fig. 3b). Moreover, the relative transcript abundance of *dgcr8* was only higher in 4WH brain (vs 1WH; *p* = 0.006) without changing in other tissues (*p* > 0.05) (Fig. 3c). Finally, the relative transcript abundance of *xpo5* was increased in 4WH brain (vs 1WH; *p* < 0.001), without changing in the other tissues (*p* > 0.05) (Fig. 3d).

### Chronic hypoxia does not inhibit m-Tor signalling in liver and white muscle

**Figure 4:**
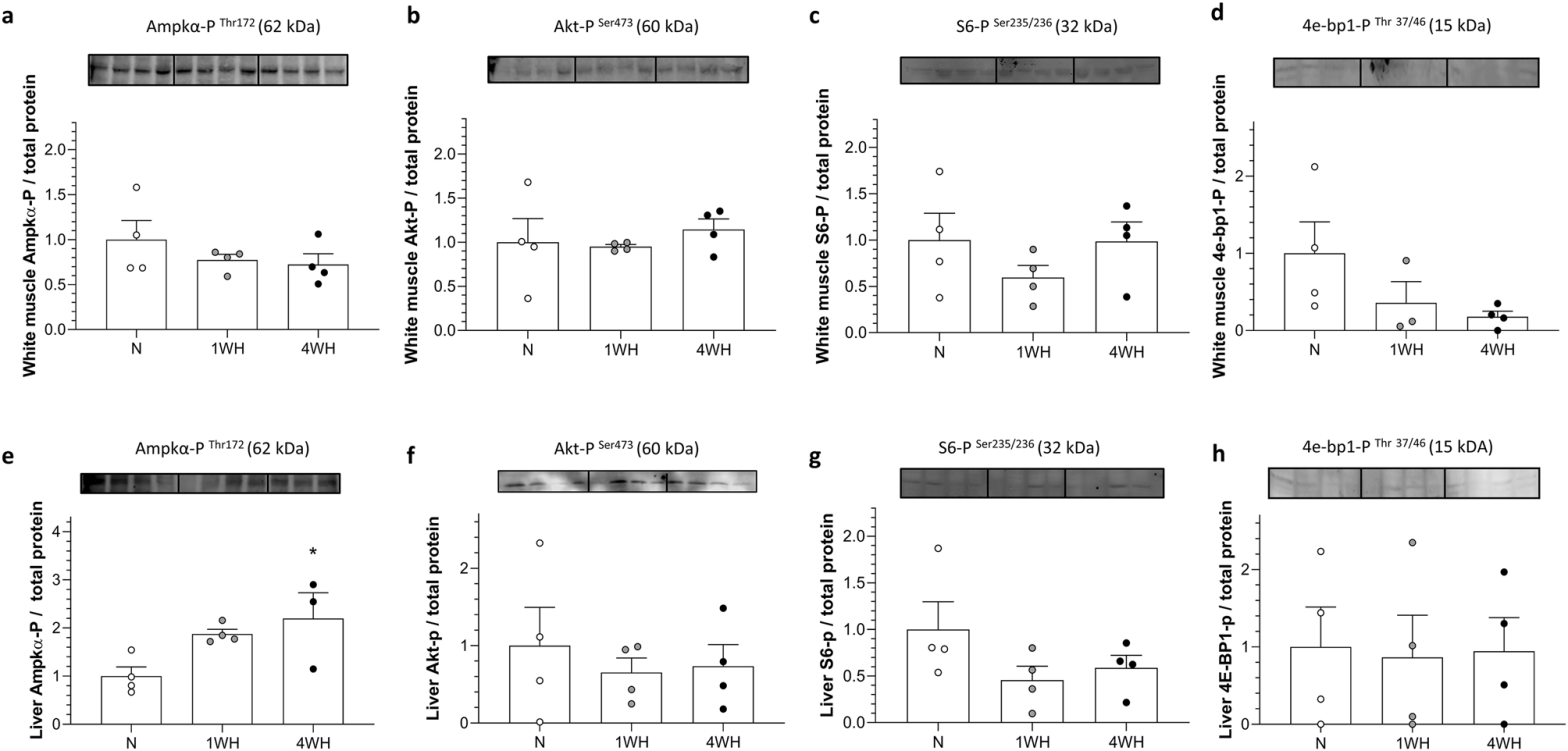
Relative abundance of phosphorylated (activated) proteins of Ampkα-P; panels (**a**) and (**e**)) and proteins involved in the m-Tor signalling pathway (Akt-P; panels (**b**) and (**f**)), S6-P; panels (**c**) and (**g**)) and 4e-bp1-P; panels (**d**) and (**h**))] in liver and white muscle of normoxic controls (N), 1-week hypoxic (1WH) and 4-week hypoxic (4WH) goldfish. Protein size, specific phosphorylation sites and Blot images for all treatment groups are provided. All targets were quantified using secondary antibody infrared-based signals and normalized to total protein stained on the membrane. Values are means ± standard error of the mean (s.e.m.); sample size = 3–4 per group. Significant effects of chronic hypoxia are indicated as *(*p* < 0.05).

Chronic hypoxia did not elicit any changes in the relative abundance of the measured phosphorylated target proteins Akt-P (liver), S6-P (liver and white muscle) and 4e-bp1-P (liver and white muscle) (*p* > 0.05) (Fig. 4b-d, f–h). However, the relative abundance of Ampkα-P increased in liver 4WH (vs normoxia; *p* = 0.04) (Fig. 4e) without changing in white muscle (*p* > 0.05) (Fig. 4a).

### Chronic hypoxia affects cholesterol metabolism transcripts in a time- and tissue-specific manner

**Figure 5:**
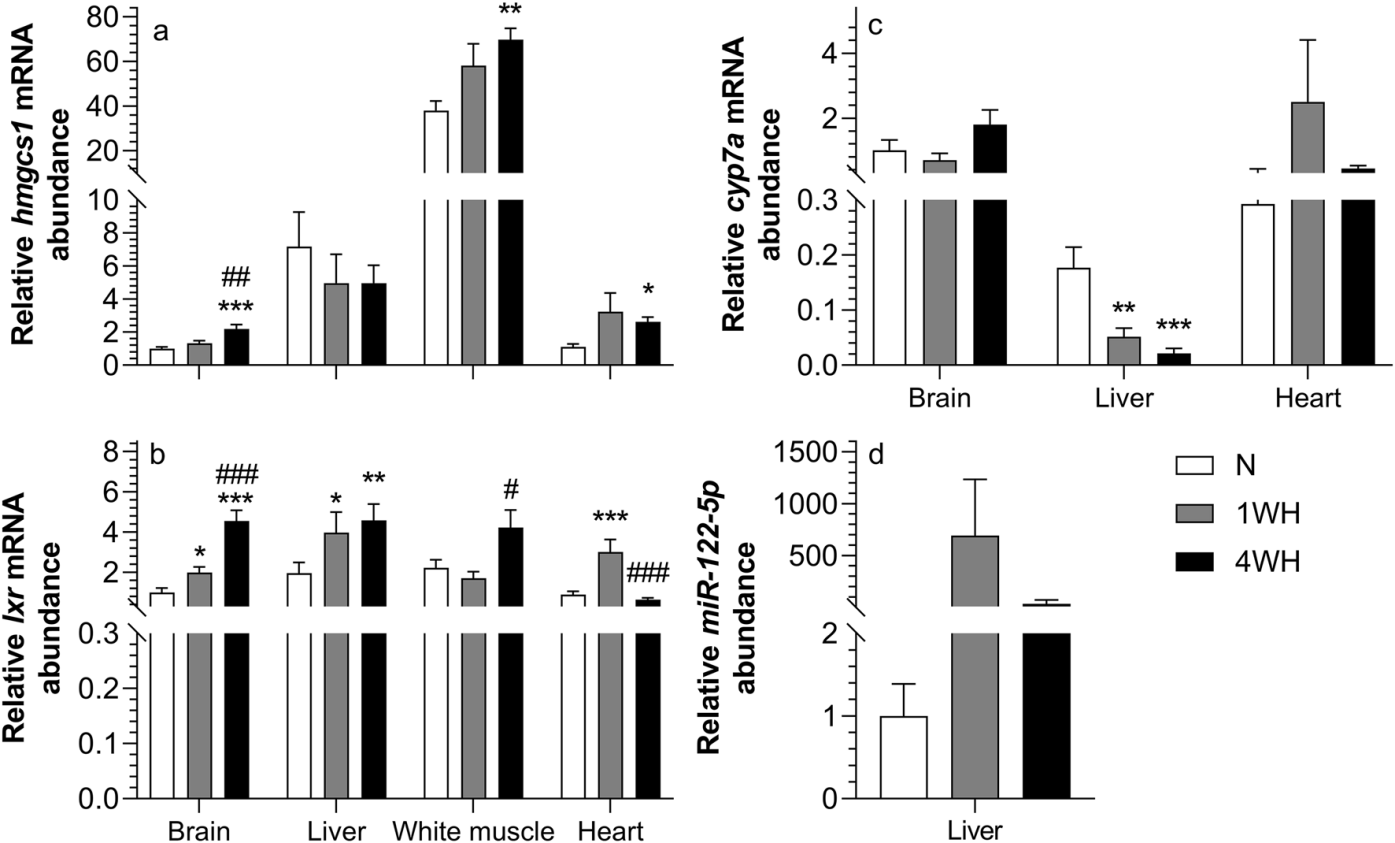
Relative transcript abundance of genes involved in cholesterol biosynthesis ((**a**) *hmgcs1*; (**b**) *lxr*; (**c**) *cyp7a*, (**d**) *miRNA-122-5p*) in tissues of normoxic controls (N), 1-week hypoxic (1WH) and 4-week hypoxic (4WH) goldfish. Values are means ± standard error of the mean (s.e.m.); sample size = 7–12 per group. Differences between normoxia and chronic hypoxia are indicated as *(*p* < 0.05), **(*p* < 0.01) and ***(*p* < 0.001). Differences between 1 and 4WH are indicated as ##(*p* < 0.01) and ###(*p* < 0.001).

The relative transcript abundance of *hmgcs1* was higher in 4WH brain (vs normoxia; *p* < 0.001 and 1WH; *p* = 0.005), white muscle (vs normoxia; *p* = 0.005) and heart (vs normoxia; p < 0.024) without changing in liver (*p* > 0.05) (Fig. 5a). Moreover, relative transcript abundance of *lxr* was increased in 4WH brain (vs normoxia and 1WH; *p* < 0.001), liver (vs normoxia; *p* = 0.007) and white muscle (vs 1WH; *p* < 0.014) but was lower in heart (vs normoxia and 1WH; *p* < 0.001). Relative transcript abundance of *lxr* in 1WH was also higher in brain (*p* = 0.023), liver (*p* = 0.048) and heart (*p* < 0.001) when compared to normoxia without changing in white muscle (*p* > 0.05) (Fig. 5b). Additionally, the relative transcript abundance of *cyp7a* was lowered in both 1WH (*p* = 0.007) and 4WH (*p* < 0.001) liver (vs normoxia) without changing in the other tissues (*p* > 0.05) (Fig. 5c). Finally, the relative transcript abundance of *miRNA-122-5p* remained unaffected by chronic hypoxia in liver (*p* > 0.05) (Fig. 5d).

### Chronic hypoxia affects abundance of transcript involved in mitochondrial dynamics and β-oxidation

**Figure 6:**
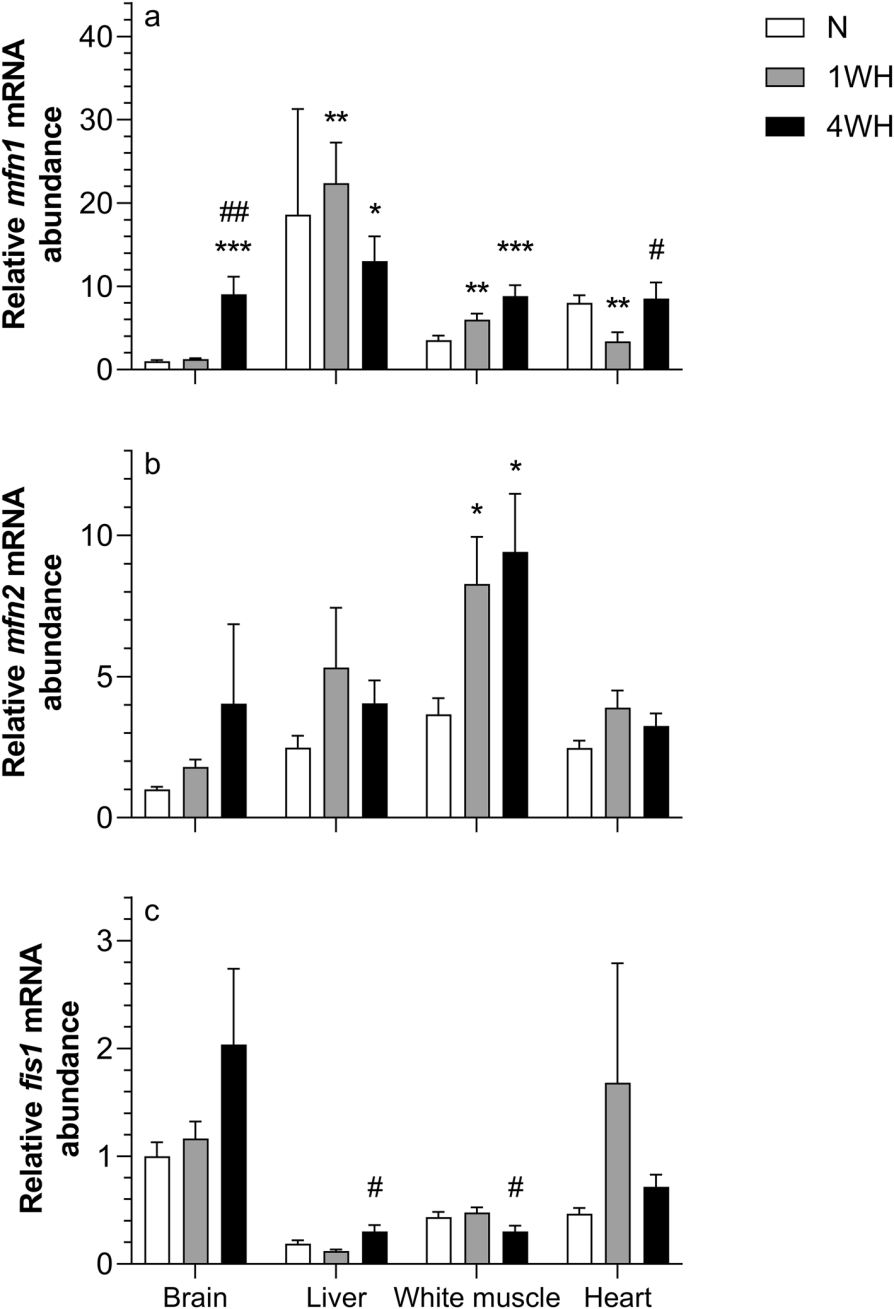
Relative transcript abundance of mRNA targets involved in mitochondrial fission and fusion ((**a**) *mfn1*; (**b**) *mfn2*; (**c**) *fis1*) in tissues of normoxic controls (N), 1-week hypoxic (1WH) and 4-week hypoxic (4WH) goldfish. Values are means ± standard error of the mean (s.e.m.); sample size = 7–12 per group. Differences between normoxia and chronic hypoxia are indicated as *(*p* < 0.05), **(*p* < 0.01) and ***(*p* < 0.001). Differences between 1 and 4WH are indicated as #(*p* < 0.05).

**Figure 7:**
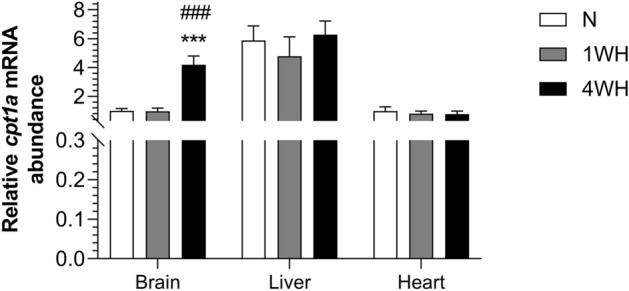
Relative transcript abundance of *cpt1a*, whose protein product is involved in the mitochondrial β-oxidation pathway, in tissues of normoxic controls (N), 1-week hypoxic (1WH) and 4-week hypoxic (4WH) goldfish. Values are means ± standard error of the mean (s.e.m.); sample size = 7–12 per group. Differences between normoxia and chronic hypoxia are indicated as ***(*p* < 0.001). Differences between 1 and 4WH are indicated as ###(*p* < 0.001).

Relative transcript abundance of *mfn1* was higher in 4WH brain (vs normoxia; *p* < 0.001 and 1WH; *p* = 0.008), liver (vs normoxia; *p* < 0.037), white muscle (vs normoxia; *p* < 0.001) and heart (vs 1WH; *p* = 0.025). Moreover, relative transcript abundance of *mfn1* was higher in 1WH liver (*p* = 0.002) and white muscle (*p* = 0.009), but lower in heart (*p* = 0.007) and remained unchanged in brain (*p* > 0.05) when compared to normoxia (Fig. 6a). Relative transcript abundance of *mfn2* was only increased in 1WH (*p* = 0.023) and 4WH (*p* = 0.03) white muscle (both vs normoxia), without changing in other tissues (*p* > 0.05) (Fig. 6b). Moreover, the relative transcript abundance of *fis1* was higher in 4WH liver (*p* = 0.025), but lower in white muscle (*p* = 0.01) (both vs 1WH) without changing in other tissues (*p* > 0.05) (Fig. 6c). The relative transcript abundance of *cpt1a* was only increased in 4WH brain (vs normoxia and 1WH; *p* < 0.001), without changing in liver and heart (*p* > 0.05) (Fig. 7).

## Discussion

This study investigates the effects of chronic hypoxia on expression of goldfish genes involved in: (i) the oxygen-sensing machinery, (ii) transcription/translation via DNA methylation dynamics and miRNA biogenesis, and (iii) cholesterol biosynthesis, mitochondrial fusion and fission, and β-oxidation. Post-translational activation of the energy sensor Ampk and m-Tor-dependent translational machinery involved in protein synthesis was also characterized. Results show that components of oxygen-sensing are robustly activated across tissues irrespective of chronic hypoxia duration except in 4WH brain. In all tissues, we also report the induction of transcripts for enzymes involved in DNA methylation turnover by chronic hypoxia exposure. In highly oxidative brain and heart tissue, global DNA hypermethylation along with partial activation of the miRNA biogenesis pathway was observed following 4WH exposure. These responses suggest that the reported DNA hypermethylation and induction of a key component of the RNA-induced silencing complex (RISC) may translate to (post-)transcriptional repression of gene transcription and protein translation especially in the chronic hypoxia-acclimated brain and heart. Future studies profiling DNA methylation status of specific promotors and bodies of genes are thus warranted to further link epigenetic repression to energetically costly cellular processes. For example, gene specific hypermethylation and/or miRNA-dependent post-transcriptional inhibition could support the reported downregulation of Na^+^/K^+^-ATPase activity^23^, the reduced transcript abundance of components of the excitatory glutamate system^26^ or components of the transcription machinery in the brain^27^. Chronic hypoxia does not suppress the m-Tor signalling pathway in white muscle or liver, which suggests that these tissues do not lower protein synthesis-dependent cellular energy expenditure via this pathway. Additionally, molecular evidence from transcripts coding for enzymes involved in cholesterol metabolism support the membrane cholesterol responses to chronic hypoxia previously shown in white muscle and brain^20^. Finally, changes in lipid metabolism and mitochondrial transcripts support an increased reliance on lipid oxidation in the brain along with an increase in mitochondrial fusion in all tissues. These results also support the higher mitochondrial respiration rates [(non-phosphorylating respiration (LEAK) and OXPHOS] previously observed in the chronic hypoxia acclimated goldfish brain^23^.

Additional molecular and physiological mechanisms contributing to the reduction in (oxygen-dependent) ATP-demand as well as the facilitation of oxygen delivery have been described in the brains of goldfish and crucian carp exposed to severe hypoxia and/or anoxia^28^. A reduction in excitatory glutamate signaling and induction of inhibitory GABA-signaling have been reported in both species^29,30^. Brain glycogen stores reveal seasonal patterns in crucian carp supporting a crucial role as a limiting energy source to promote anaerobic metabolism^31,32^. Regarding the facilitation of oxygen delivery, high levels of neuroglobin and induction of myoglobin in the brain of crucian carp^33^, as well as the maintenance of increased blood flow to the brain which contrasts with reported observations in hypoxia-tolerant ectotherms^34^ have been reported. Behavioural responses relating to both the reduction of energy expenditure and maximizing oxygen intake have equally been demonstrated, exemplified by reductions in sensory function and activity^35–37^ and increases in air gulping behaviour^38^, respectively. In sum, the brain of hypoxia-tolerant cyprinids is unique compared to other hypoxia tolerant ectotherms in that it remains active, albeit at reduced levels^39^. Regarding the heart, a strong maintenance of function has been reported in anoxic crucian carp, suggesting an important function in distribution of oxygen and glucose under severe hypoxia or anoxia unique in vertebrates^40^. Other tissues are crucially involved in mediating severe hypoxia tolerance in both species. Goldfish hemoglobin has a very high affinity for oxygen^38^ and gill surface area has been reported to expand in both crucian carp and goldfish in response to severe hypoxia^41,42^. Muscle tissue has been shown to play an important role in anaerobic waste-product removal in goldfish and crucian carp in anoxic conditions, as lactate is converted to ethanol which is subsequently excreted via the gill^43^.

In our study, chronic hypoxia induced the most pronounced and dynamic changes in marks and machinery transcripts in highly oxidative brain and heart tissues. In both tissues 4 WH exposure induced global hypermethylation and induced transcript abundance of the RISC component *ago2* whose protein product mediates miRNA-dependent post-transcriptional repression of gene expression^44^ compared to normoxic controls. Overall, this study reveals that 4WH acclimated goldfish promote metabolic suppression and hypoxia tolerance via epigenetic and post-transcriptional modifications, especially in highly oxidative tissues. These findings are in line with recent reports in other hypoxia-tolerant ectotherm vertebrates, in which epigenetic modifications in response to severe hypoxia and/or anoxia have been reported^45,46^. Future manipulative studies using pharmacological or genetic manipulation of epigenetic machinery activity and expression are now clearly warranted to directly link observed global epigenetic changes to the previously described tissue-specific contributions, especially of brain and heart, to severe hypoxia and anoxia tolerance in cyprinids. The following section aims to place our findings in the context of these known time- and tissue-dependent mechanisms of severe hypoxia-acclimation in hypoxia-tolerant cyprinids.

Chronic hypoxia greatly influences the oxygen-sensing machinery of the goldfish (Fig. 1). Transcript abundance of an *egln3* paralogue is ubiquitously induced in 1WH and 4WH fish, with the notable exception of 4WH brain. This induction unequivocally confirms the responsiveness of the molecular oxygen-sensing machinery in chronically hypoxic goldfish. Conversely, the *egln1* paralogue could only be detected in heart where it is not induced by chronic hypoxia irrespective of exposure duration (Fig. 1c).

Oxygen-sensing is highly evolutionary conserved at the molecular level^47^ and relies on *Egln*-encoded PHD enzymes to sense O_2_ changes. Several PHD isoforms exist that allow for the extension of oxygen-sensing capacity across large ranges of O_2_ levels^9^. Under normoxic conditions, PHD-mediated hydroxylation of hypoxia-inducible factor (HIF) promotes von Hippel Lindau factor (VHL)-mediated ubiquitination resulting in protein degradation. Under hypoxic conditions, lack of substrates results in decreased hydroxylation levels in HIF, thus stabilizing the transcription factor and promoting transcriptional responses to hypoxia via hypoxia-response elements (HREs) in nuclear DNA^9^. While HIF activity is principally regulated at the protein level, PHD2 and PHD3 have been shown to be part of a feedback loop, as their genes’ (*EGLN1* and *EGLN3*) promoter regions contain HREs through which they are potently induced, possibly to compensate for reduced enzyme activity under hypoxic conditions^9^. This transcriptional regulation loop makes *EGLN*s good transcriptional markers of the molecular oxygen-sensing machinery^48^.

Comparative investigation of hypoxia-tolerant organisms has provided evidence for a role of differential regulation of the oxygen-sensing machinery^49^. The higher HIF abundance in naked mole-rats has been linked to mutations in the HIF amino acid sequence that is believed to alter protein half-life by limiting VHL-dependent ubiquitination and proteasomal degradation^50^. In hypoxia-tolerant crucian carp and goldfish, these mutations are not present (as indicated by genome-derived amino acid sequences), suggesting different molecular mechanisms. The goldfish genome is complex due to multiple evolutionary genome duplication events^51^, resulting in the presence of several paralogues of the molecular oxygen-sensing machinery. Taking advantage of the recently published goldfish genome^51^, we provide a detailed description of investigated components of the goldfish molecular oxygen-sensing repertoire using basic phylogenetic approaches. Goldfish possess 5 *egln1* (including 2 *egln1b*), 2 *egln2* and 3 *egln3* genes (Supplementary File S1 online). As expected, promoter regions up to 2000 bp upstream of the transcription start sites of quantified transcripts of the *egln1* and *egln3* genes contain HREs (Supplementary File S3 online), in line with characterized HREs in mammalian *Egln1* and *Egln3*^48,52^. A strong induction of an *egln3* paralogue in response to acute hypoxia has been recently identified in early development in Atlantic salmon^53^, suggesting that this isoform may be most responsive in teleost fishes. While outside the scope of the current study, it will be of interest to establish potential (tissue-specific) transcript abundance patterns and hypoxia-responsiveness of these paralogues and to determine whether functional redundancy of multiple paralogues translates into higher basal and induced HIF pathway activity compared to less hypoxia-tolerant fish species.

In addition to protein-coding mRNA markers, we also observe an induction of the non-coding *miRNA-210-5p* in liver but not heart. *miRNA-210-5p* is the major evolutionarily conserved hypoxia-responsive and HIF-induced ‘hypoxamiR’^9^. In line with the identification and characterization of an evolutionarily conserved HRE in the *miRNA-210* promoter region^54^, we have identified HREs in the putative promoter region of goldfish in silico (Supplementary File S3 online), suggesting a HIF-dependent induction in this species. The induction of liver *miRNA-210-5p* reinforces the chronic hypoxia-responsiveness of the oxygen-sensing machinery in goldfish (Fig. 1b). This reveals that the response includes post-transcriptionally acting molecular epigenetic mechanisms. This finding further contributes to the recently reported comparative analysis of *miRNA-210-5p* regulation in hypoxia-tolerant species and suggests that tissue-specific differences and/or duration and severity of hypoxia may be linked to the observed differences in *miRNA-210-5p* induction between different hypoxia-tolerant species^14^.

The oxygen-sensing machinery is intricately linked to both transcriptional^55,56^ and post-transcriptional^14^ epigenetic mechanisms. Here we have profiled global DNA methylation as well as transcript abundance of key enzymes involved in de novo DNA methylation and demethylation to assess global changes of epigenetic machinery linked to transcriptional regulation. To evaluate potential changes in epigenetic pathways involved in post-transcriptional gene regulation, we have quantified the transcripts of key enzymes involved in canonical miRNA biogenesis.

Global DNA methylation displays a biphasic response in highly oxidative tissues and is most pronounced in the brain: following an initial hypomethylation in 1WH, hypermethylation is later detected in this tissue (Fig. 2d). In the heart, this pattern translates into significant DNA hypermethylation in 4WH vs 1WH treatment group. Our findings are in line with reports of global DNA hypermethylation when several human and murine cell lines are exposed to prolonged hypoxia in vitro and in hypoxic tumor xenografts in vivo^57–59^, but contrast with studies reporting global hypomethylation in scleroderma fibroblasts in vitro^60^. The relative transcript abundance of the *dnmt3* gene increases exclusively in the heart, suggesting a possible contribution of newly established methylation marks in response to chronic hypoxia (Fig. 2c). A study reporting HIF-1α chronic hypoxia-dependent induction of both *DNMT1* and *DNMT3* transcripts in conjunction with global DNA methylation in human cardiac fibroblasts further supports this mechanistic basis^61^. Briefly, the authors argued that siRNA-mediated functional inhibition of de novo DNA methyltransferase *DNMT3B* expression abolished the profibrotic response in hypoxia-exposed cardiac cells^61^. However, specific activity assays are needed to confirm that these transcript changes are linked to increased de novo DNA methylation activity in the goldfish heart.

Goldfish possess pairs of *tet1*, *tet2* and *tet3* genes (Supplementary File S2 online). Comparatively, *tet2* and *tet3* transcripts appear most abundant in highly oxidative tissues under normoxic conditions (Fig. 2a and b), a pattern opposite to that of the *egln3*. Interestingly, a recent study in zebrafish revealed negative crosstalk between PHD enzymes and TET, albeit at the protein level^62^. This provides an interesting possible mechanistic basis for these opposing expression patterns and suggests that highly oxidative tissues in goldfish have low baseline *egln* expression but high *tet* expression, possibly in line with different thresholds for the detection of hypoxia. Interestingly, and similarly to PHD3*,* TET enzymes are also oxygen-dependent, and can thus sense intracellular oxygen concentration directly^63^. This raises the possibility that especially highly oxidative tissues may also respond to hypoxia via TET enzymes, with subsequent effects on global DNA methylation. A general induction of *tet2* and, to a lesser extent *tet3,* is observed across time points and tissues in chronically hypoxic goldfish. Increases in *tet2* mRNA abundance compared to normoxic controls are detected in all 1WH tissues except the heart. This induction is either further increased or maintained in 4WH brain and liver (Fig. 2a). Abundance of *tet3* transcripts is elevated in all 4WH tissues compared to normoxic controls, except for liver. In white muscle, this induction is already detectable in 1WH (Fig. 2b). A recent hypothesis reconciling generally reported inhibition of TET activity^57^ and paradoxical increases in *TET* mRNA abundance^64,65^ across studies investigating hypoxia-dependent regulation in human tissue postulates that TET enzymes, similarly to PHD3, sense intracellular oxygen content, with a reduction of TET activity occurring in hypoxia^57^. However, and again similarly to the PHD1 and PHD3^9^, *TET* expression has been shown to be transcriptionally responsive to hypoxia via HREs^64,65^, resulting in increases in *TET* transcription to compensate for reduced TET activity. Indeed, our in silico analysis of putative upstream promoter sequences reveals several HREs in goldfish, suggesting an evolutionarily conserved regulatory mechanism (Supplementary File S3 online). It is thus possible that decreases in (unmeasured) goldfish Tet activity and subsequent cysteine hydroxy-methylation may in fact have contributed to the observed hypermethylation in highly oxidative tissues. Irrespective of the mode of action, overall hypermethylation observed especially in 4WH brain, but also heart tissue raises the interesting possibility that global hypermethylation contributes to reduced transcription and subsequent translation, two energetically costly processes.

The possibility for epigenetic silencing of gene expression in the brain is further supported by our profiling of the relative abundance of transcripts coding for components of the epigenetic machinery for canonical miRNA biogenesis, whose non-coding small RNA products are well described post-transcriptional inhibitors of mRNA stability and translation^44,66^. Indeed, a concurrent induction of miRNA biogenesis pathway components is observed almost exclusively in 4WH brain and heart (Fig. 3). 4WH goldfish brain transcripts coding for canonical miRNA biogenesis components are induced compared to normoxic controls (*ago2*; Fig. 3a) or 1WH (*dgcr8*; Fig. 3c, *xpo5*; Fig. 3d). The induction of miRNA biogenesis pathway in 4WH brain is opposite to generally reported chronic hypoxia-induced repression of canonical miRNA biogenesis in cancer cells^18,19,67^. All of the induced components are, albeit to a different degree, functionally involved in canonical miRNA maturation^68^. Thus, future studies probing the differential regulation of genome-wide miRNAs and in silico analysis of targeted mRNAs are warranted to explore potential contributions of miRNAs in post-transcriptional gene repression in the hypoxic goldfish brain and heart.

The brain is a large contributor to resting organismal energy demands in mammals and fish^69^, and it shows stronger metabolic suppression than other tissues in severely hypoxic and anoxic goldfish^70,71^. Several specific responses of the goldfish brain to chronic hypoxia promote energy savings thus reducing oxygen demand. For example, the activity of Na^+^/K^+^-ATPase is downregulated in the brain but not in other tissues, such as liver and white muscle^23^. Na^+^/K^+^-ATPase is crucially involved in maintaining neuronal membrane potential, and ‘channel arrest’ has been linked to a reduction in brain energy demands as well as hypoxia tolerance. Moreover, GABAergic signalling supresses neuronal signalling and is necessary to avoid neuronal excitotoxicity, a consequence of channel arrest in severe hypoxia^30^. These ATP-conserving mechanisms and/or prioritization of oxygen delivery to the brain appear to be highly effective in 4WH brain acclimated to chronic hypoxia, given that it is the only tissue and time point in our study in which the hypoxia marker *egln3* is not induced. Thus, it is equally clear that despite the coordinated global induction of epigenetic machinery and global hypermethylation in the brain, specific genes are also necessarily induced in 4WH to maintain key responses to low O_2_. Indeed, several studies have recently shown global and gene locus-specific DNA methylation dynamics in response to hypoxia in fish^66,72^. For example, global and gene locus specific effects of acute hypoxia have been reported in hypoxia-intolerant salmonids including rainbow trout, *Oncorhynchus mykiss* and Atlantic salmon, *Salmo salar*^73,74^. In rainbow trout exposed to 7d hypoxia (~ 50% reduction in dissolved O_2_ compared to normoxic controls) for example, a significant decrease in global DNA methylation was reported in the heart^73^.Chronic hypoxia (~ 75% reduction for 4 months) induced differential methylation linked to intra- as well as transgenerational impairments of reproduction, an energetically costly process, in gonads of male and female marine medaka, *Oryzias melastigma*^75,76^. Thus, all goldfish tissues, but especially the brain and heart, should be investigated in more detail by using comparable approaches. For example, investigating differentially-methylated regions of the genome and differential expression of the miRNAome in conjunction with transcriptome or proteome level changes could provide specific insights into time-dependent epigenetic control in chronically hypoxic goldfish tissues.

To test potential impacts of chronic hypoxia on energetically costly gene translation, we have assessed the activity of the ATP-sensing kinase Ampkα-P and on components of the m-Tor pathway at the cell signalling level. Previous work shows that Ampkα-P is responsive to reduced ATP charge in anoxic goldfish^24^. Moreover, components of the linked m-Tor pathway are known to be inhibited by hypoxia to minimize protein synthesis^77,78^. With the exception of activating AMPKα-P Thr^172^ phosphorylation in 4WH liver (Fig. 4e), no changes in site-specific activating phosphorylation status were identified in downstream components of the m-Tor pathway such as Akt-P (Fig. 4b and f) S6-P (Fig. 4c and g) and 4e-bp1-P (Fig. 4d and) or total tissue protein concentration (Supplementary File S4 online). Overall, these data are in line with the lack of unidirectional repression observed in hypoxic naked mole rats^79^. This suggests that, under the chronic hypoxia conditions tested, only hepatic ATP-energy sensing pathways are persistently activated without translating into a reduction in pathways stimulating mTor-dependent protein synthesis. Neither Ampkα-P Thr^172^ activation nor repression of m-Tor dependent activation of protein synthesis was observed in white muscle under chronic hypoxia. Together, these findings suggest that reduction of protein synthesis, at least via the m-Tor pathway does not contribute to ATP conservation in chronically hypoxic goldfish.

Chronic hypoxia modifies the lipid composition of goldfish membranes in ways that support metabolic suppression^20^. We have examined the effects of chronic hypoxia on the relative abundance of *hmgcs1* (Fig. 5a), *lxr* (Fig. 5b), *cyp7a* (Fig. 5c) and *miRNA-122-5p* (Fig. 5d). *Hmgcs1* encodes the first of two rate-limiting enzymes in the cholesterol biosynthesis pathway by catalyzing the condensation of acetyl-CoA with acetoacetyl-CoA to yield 3-hydroxy-3-methylglutaryl (HMG)-CoA^80^. Moreover, the liver-specific *miRNA-122-5p* is involved in the regulation of cholesterol biosynthesis and homeostasis and systemic *miRNA-122-5p* inhibition causes a decrease in circulating cholesterol levels^81–83^. Conversely, *lxr* plays a critical role in (i) cholesterol efflux from macrophages and foam cells to lipoprotein acceptors and/or (ii) hepatic cholesterol conversion to bile acids by regulating the relative abundance of several effector genes^84^. Among these, *cyp7a* is an indicator of cholesterol efflux because it is the first and rate-limiting enzyme in synthesizing bile acids from cholesterol^85^. Both *hmgcs1* and *lxr* are induced in 4WH brain, suggesting that both cholesterol synthesis and efflux are increased. This supports the overall maintenance of membrane cholesterol content in chronic hypoxia previously observed in this critical tissue^20^. A different response is observed in 4WH white muscle and heart that induce *hmgcs1* and maintain *lxr* (vs normoxic controls). This suggests an increase in cholesterol synthesis, but not efflux, and supports the increase in cholesterol content of white muscle membranes^20^. In the liver, a central tissue involved in organismal cholesterol homeostasis, both an induction of *lxr* and reduction of its target gene *cyp7a* are observed, suggesting that chronic hypoxia uncouples normal responses of the cholesterol degradation pathway. In contrast to protein-coding genes, the relative abundance of the evolutionarily conserved *miRNA-122-5p* is not affected by chronic hypoxia. The transcriptional regulation of these key genes involved in cholesterol metabolism clearly support reported changes in membrane cholesterol composition, a newly proposed mechanism of metabolic suppression^86^.

Mitochondrial plasticity is a generally acknowledged factor in hypoxia responses, acting to limit intracellular oxygen demand by regulating oxidative phosphorylation (OXPHOS), among other processes^47,87–89^. We have recently investigated mitochondrial plasticity in several goldfish tissues and found that fuel preference was highly tissue-dependent during chronic hypoxia^23^. Here, we have identified a general prioritization of induction of transcripts coding for proteins involved in mitochondrial fusion compared to fission, especially *mfn1* and to a lesser extent *mfn2*. While relative *mfn1* transcript abundance is induced by chronic hypoxia in liver and white muscle, its relative transcript abundance is only induced in 4WH brain (Fig. 6a). Conversely, a transient decrease is observed in 1WH heart. The induction of *mfn2* is highly tissue-specific, with elevations observed in white muscle irrespective of chronic hypoxia duration (Fig. 6b). The mitochondrial fission factor *fis1* does not reveal significant changes in its relative transcript abundance compared to normoxic controls (Fig. 6c), but a dynamic regulation characterized by higher relative transcript abundance in 4WH vs 1WH liver, and lower relative transcript abundance in 4WH vs. 1WH white muscle.

The generally observed induction of transcripts coding for mitochondrial fusion proteins across tissues in 4WH are supported by previous reports in goldfish linking general reductions in mitochondrial cytochrome *c* oxidase respiration indicating a decrease in mitochondrial density under chronic hypoxia^23^. This suggests that mitochondrial fusion under chronic hypoxia promotes mitochondrial membrane stability to protect mitochondria from damage, mitophagy and the induction of cellular apoptosis^85^. Interestingly, a recent comparative study reported *MFN1* transcript abundance to be higher in brains of hypoxic Tibetan chickens compared to hypoxia-intolerant dwarf laying chickens. The authors interpreted this finding as evidence for a role of mitochondrial fusion in mediating brain hypoxia tolerance in the Tibetan birds^90^. Thus, our results provide support for a role of activation of mitochondrial fusion pathways in goldfish hypoxia-tolerance, a finding that, however, clearly warrants further functional and histological investigation. We have also recently identified a brain-specific switch in mitochondrial substrate preference from carbohydrates to lipids in chronically hypoxic goldfish^23^. Here we have profiled the relative transcript abundance of *cpt1a*, considered the rate limiting enzyme in fatty acid oxidation, as well as genes involved in cholesterol synthesis and degradation pathways. Here, *cpt1a* is induced in 4WH brain, but not liver or heart (Fig. 7), in line with the reported increase in fatty acid metabolism of goldfish brain mitochondria exposed to the same experimental conditions^23^.

In summary, this study shows that tissue-specific differences in the relative transcript abundance of molecular oxygen-sensing machinery and linked epigenetic machinery components exist between highly oxidative and other goldfish tissues. Chronic hypoxia strongly activates the oxygen-sensing machinery of all tissues except 4WH brain, irrespective of acclimation duration. Goldfish also rely on transcriptional silencing of chronically hypoxic brain and heart via hypermethylation after being transiently hypomethylated after 1WH. This suggests that transcriptional modifications support metabolic suppression of these critical tissues but require a long hypoxia exposure to occur. Indeed, most previously described acclimation mechanisms of severe hypoxia or anoxia acclimation in goldfish and crucian brain and heart, but also other tissues have a rapid onset in the range of days^28–43^, coinciding with global DNA hypomethylation and potential widespread transcript induction in our current study. Thus, while epigenetic control of specific processes linked to chronic hypoxia in the goldfish brain (and other tissues) warrants more detailed investigation at the gene level, the global pattern is suggestive of an initial de-repression of epigenetic marks in the brain, followed by a potent global establishment of repressive epigenetic marks and induction of post-transcriptional miRNA machinery components. Together, this pattern is suggestive of an epigenetically mediated initial general transcriptional activation of underlying acclimation processes in initial stages of chronic hypoxia exposure in highly oxidative tissues, followed by transcriptional and translational inhibition in these tissues to lower energetic cost and thus oxygen use.

While we show that chronic hypoxia only activates hepatic Ampkα-P and does not repress cell signaling involved in protein synthesis of liver or white muscle, limited tissue amount precluded us from investigating regulation of protein translation pathways in highly oxidative tissues. Such studies are warranted in the future. Additional quantified gene expression changes support the membrane cholesterol responses previously reported for brain and white muscle in 4WH-exposed goldfish^20^. Finally, we show that the previously reported chronic hypoxia-induced mitochondrial plasticity and changes in lipid metabolism in goldfish are, at least in part, mediated at the transcriptional level. Specifically, we report a possible contribution of activated mitochondrial fusion pathways to chronic hypoxia tolerance and metabolic suppression and reveal that induction of *cpt1a* transcripts in the brain contributes to the reported switch in brain fuel preference to lipid oxidation in 4WH-exposed goldfish, suggesting the brain maintains sufficient oxygen delivery to support this mitochondrial process^23^. Overall, this study shows that chronic hypoxia in goldfish greatly activates molecular oxygen-sensing, with the notable exception of the brain, again suggesting prioritization of oxygen delivery and/or reduction of oxygen use in this tissue. Because induction of epigenetic markers and machinery is observed in the brain and, to some extent, the heart, our study suggests that epigenetic suppression of energetically costly transcription/translation in highly oxidative tissues may contribute to longer-term, but not initial response to hypoxia. Finally, molecular evidence provided in the current study further supports a role for membrane remodeling and mitochondrial plasticity previously reported to support metabolic suppression in chronically hypoxic goldfish^20,23^.

## Methods

### Animals

Adult common goldfish (*Carassius auratus*, Linnaeus 1758; *N* = 36) were purchased from AQUAlity Tropical Fish Wholesale (Mississauga, Ontario, Canada) and held in a 1200 L flow-through holding tank in dechloraminated, well-oxygenated water, under a 12 h:12 h light:dark photoperiod, and were fed 3 mm floating fish pellets (Profishent; Martin Mills; Elmira, Ontario, Canada) once a day. They were randomly allocated to normoxia or chronic hypoxia. All measurements were performed at 13 °C, and the fish were acclimated to this temperature for at least 2 weeks in the holding tank before starting experiments. Water was then made progressively hypoxic over 7 days by bubbling increasing amounts of N_2_ through a column filled with glass beads. Water PO_2_ was measured using galvanic oxygen probes (Loligo Systems, Tjele, Denmark). The probes were calibrated before each measurement using air-saturated (20.9% O_2_) water. Fish were randomly allocated to either normoxia, 1-week hypoxia (1WH) or 4-week hypoxia (4WH) (*N* = 12 per group). The experimental timeline for chronic hypoxia exposure in our study was based on previous literature investigating molecular and physiological mechanisms of hypoxia acclimation in fishes^40,91,92^, allowing for better comparability. The specific experimental choice to investigate tissues sampled from fish exposed to normoxia, 1WH, and 4WH was made to align these specific time-points with previously reported endpoints regarding membrane lipid composition and mitochondrial fuel selection^20,23^. Atmospheric oxygen availability was gradually reduced from normoxic (21 kPa) to chronic hypoxia (2.1 kPa) which was achieved by 1 WH and maintained to 4 WH^20,23^. During the first week of hypoxia exposure oxygen availability was gradually decreased from 21 kPa saturation (day 1) to 10.5 kPa (day 2), 8.4 kPa (day 3) , 6.3 kPa (day 4), 4.2 kPa (day 5), 3.15 kPa (day 6) finally reaching 2.1 kPa PO2 (1 WH). These experimental conditions were selected because it induces significant suppression of goldfish aerobic metabolism, but without causing any ATP synthesis from anaerobic ethanol production^20^. No mortalities were observed throughout the experiment. All procedures were approved by the Animal Care Committee of the University of Ottawa (protocol BL-1625) and adhered to the guidelines established by the Canadian Council on Animal Care for the use of animals in research. Animals were euthanized using severing of the spinal cord, and dissected tissues immediately stored at -80 °C for approximately one year prior to subsequent analyses.

### Real-time RT-PCR assays for mRNA and miRNA

#### Relative abundance mRNA quantification

Total RNA from brain, liver, white muscle and heart was extracted by homogenizing 50 mg of tissue in TRIzol reagent (Invitrogen, Burlington, ON, Canada) using a sonicator (Fisher Scientific Sonic Dismembrator model 100, San Diego, CA, USA). Extracted RNA was quantified using a NanoDrop 2000c UV–Vis Spectrophotometer (Thermo-Fisher Scientific, Mississauga, ON, Canada). Next, cDNA was generated using a QuantiTech Reverse Transcription Kit (Qiagen, Toronto, ON, Canada) following the manufacturer’s protocol which includes a DNA wipeout step before reverse transcription occurs. A noRT control was included to assess potential DNA contamination in subsequent gene expression assays. Two-step real-time RT-PCR assays were performed on a BioRad CFX96 instrument (Bio-Rad, Mississauga, ON, Canada) to quantify fold-changes in relative mRNA abundances of key transcripts involved in hypoxia-sensing (*egl-9* Family members, e*gln1*, e*gln3),* transcriptional epigenetic pathways, specifically DNA methylation (de novo DNA methyltransferase 3; *dnmt3,* ten-eleven-translocation methylcytosine dioxygenases 2 and 3; *tet2* and *tet3*) and post-transcriptional epigenetic pathways, specifically canonical miRNA biogenesis components (*dgcr8*, *dicer*, *ago2*, *xpo5*). Additionally, transcripts involved in lipid metabolism, specifically fatty acid oxidation (*cpt1a*) as well as cholesterol biosynthesis (*hmgcs1*), sensing and degradation (*lxr, cyp7a)*, and mitochondrial dynamics, specifically mitochondrial fusion (*mfn1, mfn2*) and fission (*fis1*). For each assay, a standard curve consisting of serial dilutions of pooled cDNA samples were run in duplicate for each experiment. The total reaction volume was 20 μl, which consisted of 1 μl of diluted cDNA template, 1 μl of 10 nM specific forward and 1 μl of 10 nM specific reverse primer (Supplementary File S5 online), 10 μl of SsoAdvanced Universal Inhibitor-Tolerant SYBR Green Supermix (Bio-Rad), and 7 μl of H_2_O for each individual reaction. Real-time RT-PCR cycling parameters were a 5 min activation step at 95 °C, followed by 40 cycles consisting of a 20 s denaturation step at 95 °C and a 30 s annealing and extension step at a primer-specific temperature (Supplementary Files S1-S3, online). NoRT and no template controls were included in each assay to control for DNA contamination. After each run, melting curves were produced and monitored for single peaks to confirm the specificity of the reaction and the absence of primer dimers. All amplification efficiencies calculated from serially diluted 7-point standard curves were between 86.8–110%, with R^2^ values > 0.91 (Supplemental File S5 online). For several transcripts, low abundance in specific tissues precluded the creation of standard curves and thus the determination of relative-fold change between N and 1WH and 4WH. Relative transcript abundance derived from standard curves was normalized using the NORMA-Gene approach as described by Heckman et al.^93^.

#### Relative miRNA abundance quantification

Total RNA obtained from the previously described TRIzol Reagent procedure (see Sect. 2.2.1) was used to synthesize cDNA with HiFlex buffer using the miScript II RT kit (Qiagen, Toronto, ON, Canada) according to the manufacturer’s instructions. Specific miRNAs were subsequently quantified using the miScript SYBR Green PCR kit (Qiagen) with miRNA-specific forward primers and a universal reverse primer. A standard curve consisting of serial dilutions of pooled cDNA, as well as a negative no-RT control consisting of cDNA generated in a reaction that did not include reverse transcriptase was run in duplicate. Reactions were run on a CFX96 instrument (Bio-Rad) with a total volume of 20 μl containing 1 μl cDNA, 1 μl of 10 μM miRNA specific forward primer, 1 μl miScript Universal Reverse Primer, 10 μl 2 × QuantiTect SYBR Green PCR Master Mix (Qiagen), and 7 μl H_2_O, according to the manufacturer’s instructions. Cycling parameters were a 15 min 95 °C activation step, followed by 40 cycles of 15 s incubation at 94 °C, 30 s at a primer-specific annealing temperature 55–60 °C, and 30 s at 70 °C. After each assay run, specific melting curves were produced by a gradual increase in temperature from 65 °C to 95 °C in 0.5 °C increments every 5 s. NoRT and no template controls were included in each assay to control for DNA contamination. The final curves were monitored for single peaks to confirm the specificity of the reaction and the absence of primer dimers. Relative transcript abundance derived from standard curves was normalized by the NORMA-Gene approach as described by Heckmann and colleagues^93^.

### Global DNA methylation

Relative levels of global DNA methylation (%) was assessed using the MethylFlash Global DNA Methylation (5-mC) ELISA Easy Kit, Colorimetric (Source: Epigentek, Cat # P-1030), according to manufacturer’s instructions. This kit quantifies global DNA methylation levels by measuring levels of 5-methylcytosine (5-mC) colorimetrically in an ELISA assay using genomic DNA. Briefly, genomic DNA was extracted from the Trizol group collected for brain liver, white muscle and heart tissues in Sect. 2.2.1 of normoxic,1-week hypoxic (1WH) and 4-week hypoxic (4WH) goldfish. Briefly, the DNA phase previously collected was precipitated using 100% ethanol and then washed twice using 0.1 M sodium citrate in 10% ethanol. After centrifuging, the collected pellet was suspended in 8 mM NaOH to solubilize the DNA before determining the DNA yield (Source: Invitrogen, Burlington, ON, Canada, Cat # 15,596,026 and 15,596,018). DNA was then incubated with 100 μl of binding solution in a 96-well microplate for 60 min at 37 °C. A negative control representing unmethylated polynucleotide containing 50% of cytosine, and positive control representing methylated polynucleotide containing 50% 5-methylcytosine were also loaded into independent wells on the same microplate. The wells were then incubated for 60 min at room temperature with a capture antibody (1 ng ml^-1^). Following the incubation period, the binding solution was removed, and each well washed three times with diluted washing buffer. Subsequently, a 50 μl aliquot of 5-mC detection complex antibody solution cocktail (1 μl of mC antibody + 1 μl of signal indicator + 1 μl of enhancer solution in 1 ml of diluted washing buffer) was added and incubated at room temperature for 50 min. This solution complex was then removed before washing each well with diluted washing buffer 5 times. Following that, 100 μl of developer solution was added to each well simultaneously and incubated at room temperature for 3 min until the developer solution turned blue. Finally, 100 μl stop solution was added to each well to halt the reaction and then absorbance values were read using a Spectra Max Plus384 Absorbance Microplate Reader (Molecular Devices, Sunnyvale, CA) at 450 nm.

### Western blotting

Frozen liver and white muscle from the normoxic and 1WH and 4WH goldfish groups (*N* = 4 per group) were homogenized on ice with a sonicator (Fisher Scientific Sonic Dismembrator model 100, San Diego, CA) in 400 μl of buffer per 100 mg of tissue. During homogenization, samples were kept in a buffer containing 150 mmol/l NaCl, 10 mmol/l Tris, 1 mmol/l EGTA, 1 mmol/l EDTA (pH 7.4), 100 mmol/l sodium fluoride, 4 mmol/l sodium pyrophosphate, 2 mmol/l sodium orthovanadate, 1% (vol/vol) Triton X-100, 0.5% (vol/vol) NP40-IGEPAL, and a protease inhibitor cocktail (Roche, Basel, Switzerland). Homogenates were centrifuged at 15,000 g for 5 min at 4 °C, and the resulting supernatants were recovered and stored at − 80 °C. Protein concentrations were determined using bicinchoninic acid (BCA) assay (B9643, Sigma-Aldrich) with BSA as standard. A denaturing, nonreducing SDS-PAGE was used to separate proteins. Lysates were diluted in the previously described buffer containing protease inhibitor for a total of 30 μg of total protein for liver and 50 μg of total protein for white muscle in 15 μl before 15 μl of 2 × Laemmli buffer were added for a total loading volume of 30 μl. The prepared samples were denatured at 95 °C for 5 min and quick chilled on ice before loading on the gel. Gels were cast as 10% resolving gel consisting of 5 ml ddH20, 2.5 ml buffer B pH 8.8 (1.5 M Tris base, 0.04% SDS at pH 8.8; both BioShop, Burlington, ON Canada) dissolved in dH_2_O, 2.5 ml 40% acryl/Bis (Bio-Rad, Mississauga, ON, Canada) and polymerized with 50 μl 10% APS (Sigma-Aldrich Oakville, ON, Canada) and 20 μl TEMED (Life Technologies Burlington, ON, Canada), and a 4% stacking gel [consisting of 3.25 ml ddH_2_O, 1.25 ml buffer C pH 6.8 (0.5 M Tris, 0.04% SDS dissolved (BioShop) in dH_2_O, 0.5 ml 40% acryl/bis polymerized with 25 μl 10% APS, and 10 μl TEMED]. Gels were immersed in 1 × Tris glycine SDS (TGS) running buffer, consisting of Tris base 2.5 mM, glycine 0.192 M, and 0.1% SDS (all BioShop Canada) dissolved in dH_2_O, and samples were loaded with 5 μl of Page Ruler prestained protein ladder (Thermo Fisher, Ottawa, ON, Canada).

Proteins were migrated in the gel at 100 V for ~ 2 h. After migration, they were blotted onto nitrocellulose 0.45-mm pore size membrane paper (Millipore, Etobicoke, ON, Canada) by wet transfer using the Mini TransBlot system (Bio-Rad) with blotting buffer (250 mM Tris base, 1920 mM glycine; all BioShop Canada) dissolved in dH2O, by applying 100 V for 2 h. Membranes were incubated with Odyssey blocking buffer (LI-COR Biosciences Lincoln, NE) for 1 h at room temperature using an orbital shaker. After the blocking step was completed, membranes were cut based on the molecular weight marker to allow separate development of post-translationally modified Akt-P^Ser473^, S6-P^Ser235/236^, 4e-bp1-P^Thr^^37^^/^^46^ and Ampkα-P^Thr172^ proteins with specific primary antibodies validated in fish^82,94^. Partial membranes containing the relevant molecular weight range of proteins were incubated with rabbit raised primary Akt-P (no. 9271), S6-P (no.2211) 4e-bp1-P (no.9459) or Ampkα-P (no.2532) antibodies (Cell Signaling Technology Ozyme, Saint Quentin Yvelines, France), respectively, at a concentration of 1:10,000 on an orbital shaker at 4 °C overnight. Membranes were washed four times for 5 min with PBS + 0.1% Tween 20 (Sigma-Aldrich) then incubated with an IRDye Infrared dye (680 nm coupled) secondary goat anti-rabbit IgG antibody (LI-COR Biosciences). Bands were visualized by infrared fluorescence using the Odyssey Imaging System (LI-COR Biosciences) and quantified by Odyssey Infrared imaging system software (v.3.0; LI COR Biosciences). p-Akt, S6-P and 4e-bp1-P protein intensity were normalized to Blot total protein stain intensity measured using the Revert ™ 700 Total Protein stain kit (no.926–11,010, LI COR Biosciences) and expressed as relative-fold change compared with control groups for liver and white muscle.

### Statistics

Statistical analyses were performed using GraphPad Prism 8.3.1. Data were analyzed using a one-way analysis of variance (ANOVA) for all experiments to test for the significant effects of chronic hypoxia treatment, followed by the Holm-Sidak post-hoc test for multiple comparisons. Normality was assessed using the Shapiro–Wilk test and homoscedasticity by the Levene’s test. When the assumptions of normality or equality of variances were not met, the data were normalized by log10 or square root. If transformation was unsuccessful, non-parametric Kruskal–Wallis one-way ANOVA on ranks test was performed, followed by Dunn’s post-hoc test for multiple comparisons. All values presented are means ± s.e.m., and a level of significance of *P* < 0.05 was used in all tests.

### Ethics declarations

All procedures were approved by the Animal Care Committee of the University of Ottawa and adhered to the guidelines established by the Canadian Council on Animal Care for the use of animals in research. The study is reported in accordance with the ARRIVE guidelines (https://arriveguidelines.org).

## Supplementary Information

Supplementary Information.

## Data Availability

The datasets generated during and/or analysed during the current study are available from the corresponding author on reasonable request.
