# Supplementary material for: Epigenetic and post-transcriptional repression support metabolic suppression in chronically hypoxic goldfish
Source: Sci Rep. 2022 Apr 2;12:5576. doi: 10.1038/s41598-022-09374-8 (PMC8976842; doi:10.1038/s41598-022-09374-8)

**Supplementary File S1**. Diversity of goldfish Egln enzymes relevant to the molecular hypoxia-sensing pathway. Predicted amino acid sequences for Egln isoforms and paralogues were extracted from the NCBI-deposited genome sequences of goldfish (ID: 10773) as well as zebrafish (ID: 50), human (ID: 51) and mouse (ID: 52). Maximum-likelihood-based tree phylogenies were constructed using the standard parameters of the Phylogeny.fr database 1 and branch support calculated using the a LRT statistical test ^1^.

1 Dereeper, A. *et al.* Phylogeny.fr: robust phylogenetic analysis for the non-specialist. *Nucleic Acids Research* **36**, W465-W469, doi:10.1093/nar/gkn180 (2008).

**Supplementary File S2**. Diversity of goldfish Tet dioxygenase enzymes relevant to the molecular hypoxia-sensing pathway. Predicted amino acid sequences for Tet isoforms and paralogues were extracted from the NCBI-deposited genome sequences of goldfish (ID: 10773) as well as zebrafish (ID: 50), human (ID: 51) and mouse (ID: 52). Maximum-likelihood-based tree phylogenies were constructed using the standard parameters of the Phylogeny.fr database ^1^ and branch support calculated using the a LRT statistical test ^1^.

1 Dereeper, A. *et al.* Phylogeny.fr: robust phylogenetic analysis for the non-specialist. *Nucleic Acids Research* **36**, W465-W469, doi:10.1093/nar/gkn180 (2008).

**Supplementary File S3**. Putative promoter sequences (2000 bp upstream of TSS) were extracted for goldfish *egln* and *tet* genes from NCBI, and manually screened for HRE consensus sequences (highlighted) to infer possible HIF-binding capacity.


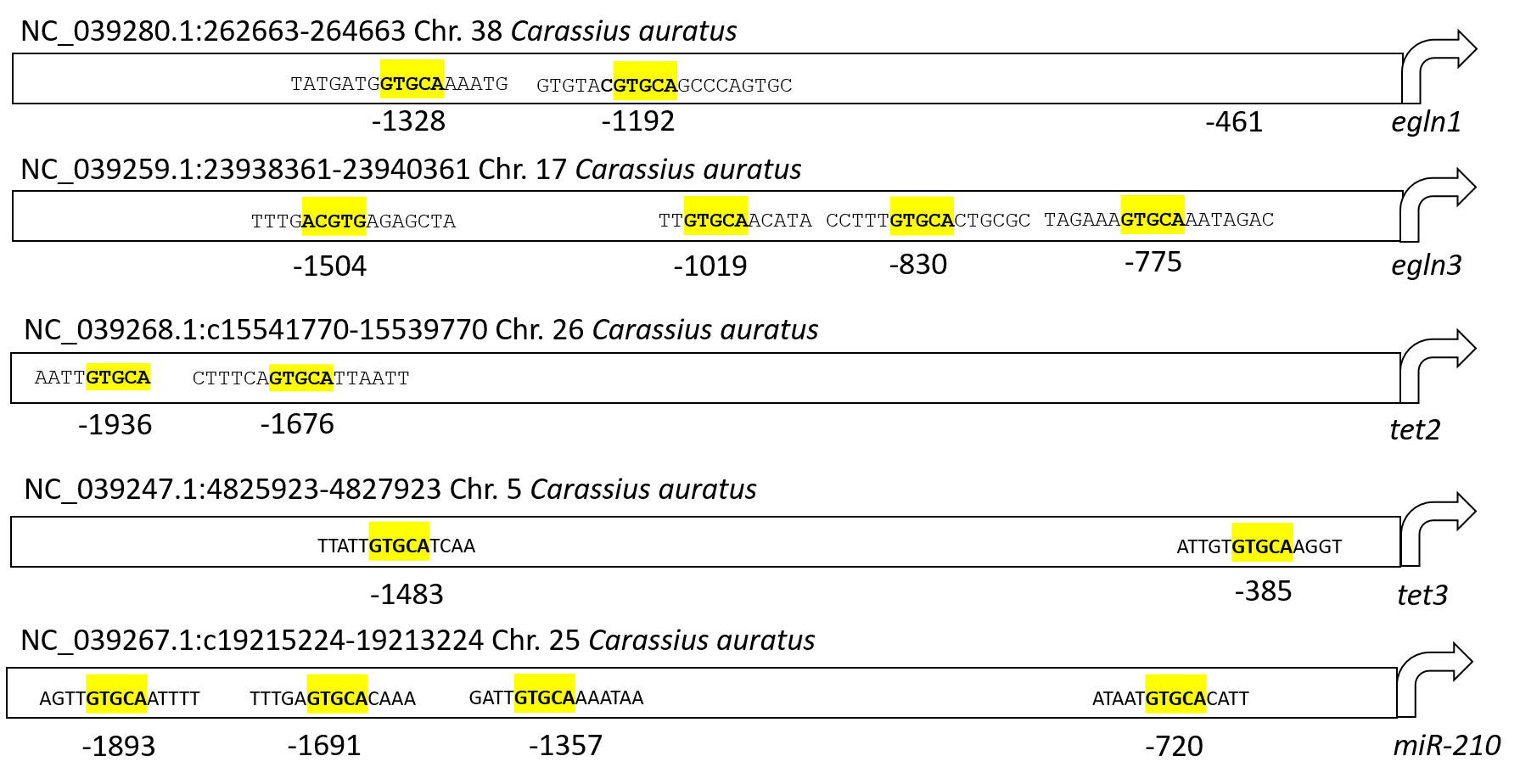


**Supplementary File S4**. Expression of total protein in white muscle (a) and liver of normoxic controls (N), 1-week hypoxic (1WH) and 4-week hypoxic (4WH) goldfish. Total protein was used to normalize all western blot data (see Fig. 4 in manuscript).


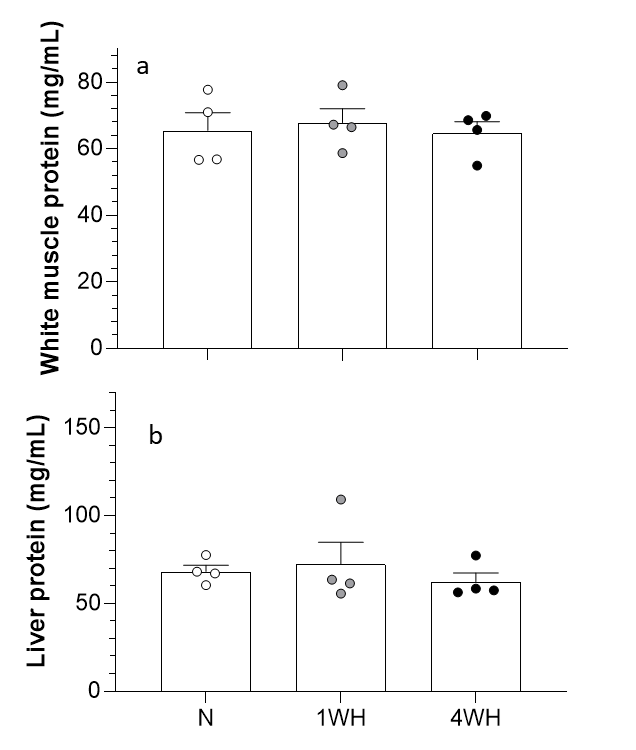


**Supplementary File S5**. *Real-time* RT-PCR primer sequences and reaction parameters of gene targets involved in oxygen sensing (*egln1*, *egln3* and *miR-210-5p*), DNA methylation dynamics (*tet2*, *tet3* and *dnmt3*), miRNA biogenesis pathway (*dgcr8*, *dicer*, *xpo* and *ago2*), cholesterol biosynthesis (*hmgcs1, lxr* and *cyp7a* and *miR-122-5p*), β-oxiation (*CPT1a*) and mitochondrial fission and fusion (*mfn1*, *mfn2* and *fis1*).

**Supplementary File S6**. Western Blot source images obtained from white muscle (A-D) and liver (E-H) samples for specific phosphorylated proteins involved in energy sensing and mTor-dependent protein synthesis pathway regulation. Experimental groups are designated as follows: Normoxia (N), one week hypoxia (1WH), four weeks hypoxia (4WH).


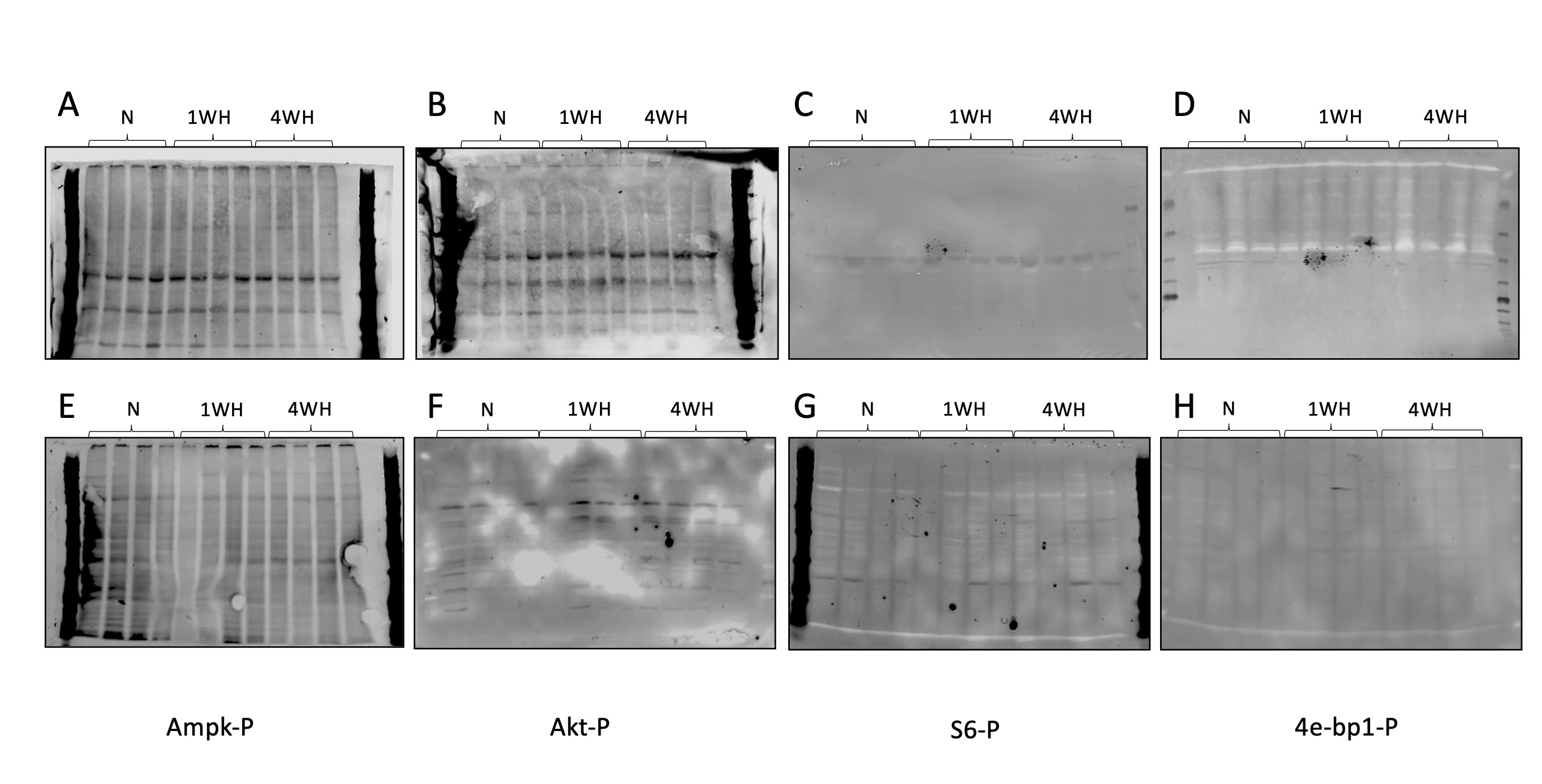

Supplement: Supplementary file 1 — Supplementary Information. [file 41598_2022_9374_MOESM1_ESM.docx]
